# Supplementary material for: The ALPK1/TIFA/NF-κB axis links a bacterial carcinogen to R-loop-induced replication stress
Source: Nat Commun. 2020 Oct 9;11:5117. doi: 10.1038/s41467-020-18857-z (PMC7547021; doi:10.1038/s41467-020-18857-z)
Supplement: Supplementary file 1 — Supplementary Information [file 41467_2020_18857_MOESM1_ESM.pdf]

The ALPK1/TIFA/NF-κB axis links a bacterial carcinogen to R-loop-induced replication stress

Bauer/Nascakova/Mihai et al., Nature Communications 2020

Supplementary Information

Supplementary Figures 1-8

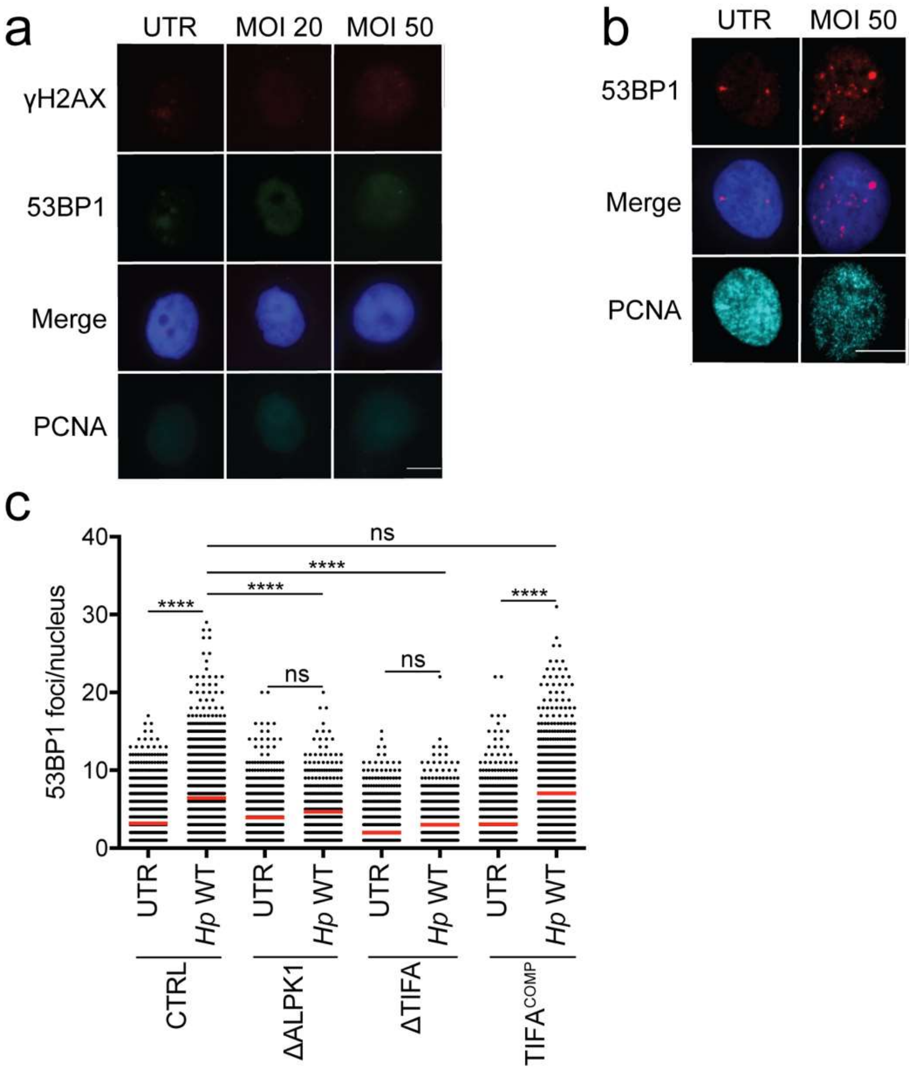

Supplementary Fig. 1. ***H. pylori*** **induces DNA DSBs in gastric epithelial cells that depend on a functional ALPK1/TIFA signaling axis and occur in S-phase. a** Wild type AGS cells were infected for 6 hours with *H. pylori* strain P12 at MOIs of 20 and 50 and subjected to immunofluorescence staining for phosphorylated H2AX (γH2AX), 53BP1 and PCNA as well as DAPI. Images of $PCNA^-$ cells are representative of three independently conducted experiments. **b** Wild type AGS cells were infected for 6 hours with *H. pylori* strain G27 at MOI 50 and stained for 53BP1 and PCNA as well as DAPI. Images of $PCNA^+$ cells are representative of three independently conducted experiments. Scale bar in a and b, 10 µm. **c** The indicated mutant cell lines in the AGS background were infected for 6 hours with *H. pylori* strain P12 at MOI 50 and subjected to immunofluorescence staining for phosphorylated H2AX (γH2AX), 53BP1 and PCNA as well as DAPI. Scatter dot plots of >811 and up to 2424 PCNA-positive cells per condition are shown. Data are pooled from three independent experiments. P-values in c were calculated by one-way ANOVA with Dunn's multiple comparisons correction; ns, not significant; $^{****}p < 0.0001$. Note that the ALPK1 ko cell line shown here was generated independently of the one shown in Figure 1.

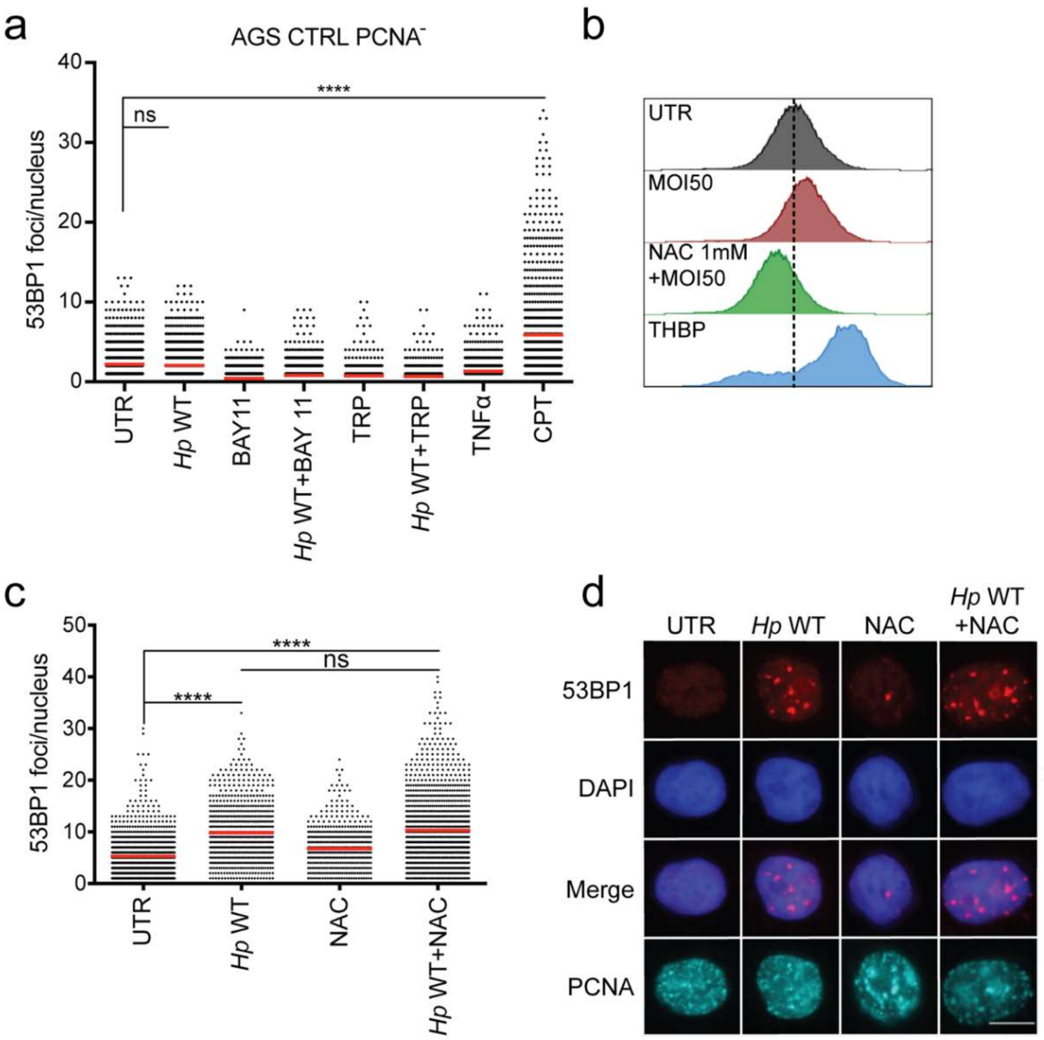

Supplementary Fig. 2. *H. pylori*-induced DNA DSBs are specific to S-phase cells and not caused by reactive oxygen species. a AGS cells were infected for 6 hours with *H. pylori* P12 at an MOI of 50 in the presence or absence of the NF-κB inhibitor BAY 11-7082 and the transcription inhibitor triptolide (1 μM and 100 nM final concentration, respectively), or were treated with 10 nM TNF-α or 100 nM camptothecin (CPT) for 6 hours, and subjected to immunofluorescence staining for 53BP1 and PCNA as well as DAPI. Scatter dot plots of >422 and up to 903 PCNA-negative cells per condition are shown. Data are pooled from three independent experiments. **b-d** AGS cells were infected for 6 hours with *H. pylori* P12 at an MOI of 50 in the presence or absence of N-acetyl-cysteine (NAC) at 1 mM final concentration. ROS formation as detected by flow cytometry of DCFDA-stained (FITC) cells is shown in b (treatment with 50 μM Tert-Butyl Hydrogen Peroxide -TBHP- for 6 hours served as positive control) and scatter dot plots of >895 and up to 1110 PCNA-positive cells per condition are shown in c alongside representative images in d (scale bar 10 μm). Data in c are pooled from three independent experiments. P-values in a and c were calculated by one-way ANOVA with Dunn's multiple comparisons correction; ns, not significant; ****$p < 0.0001$.

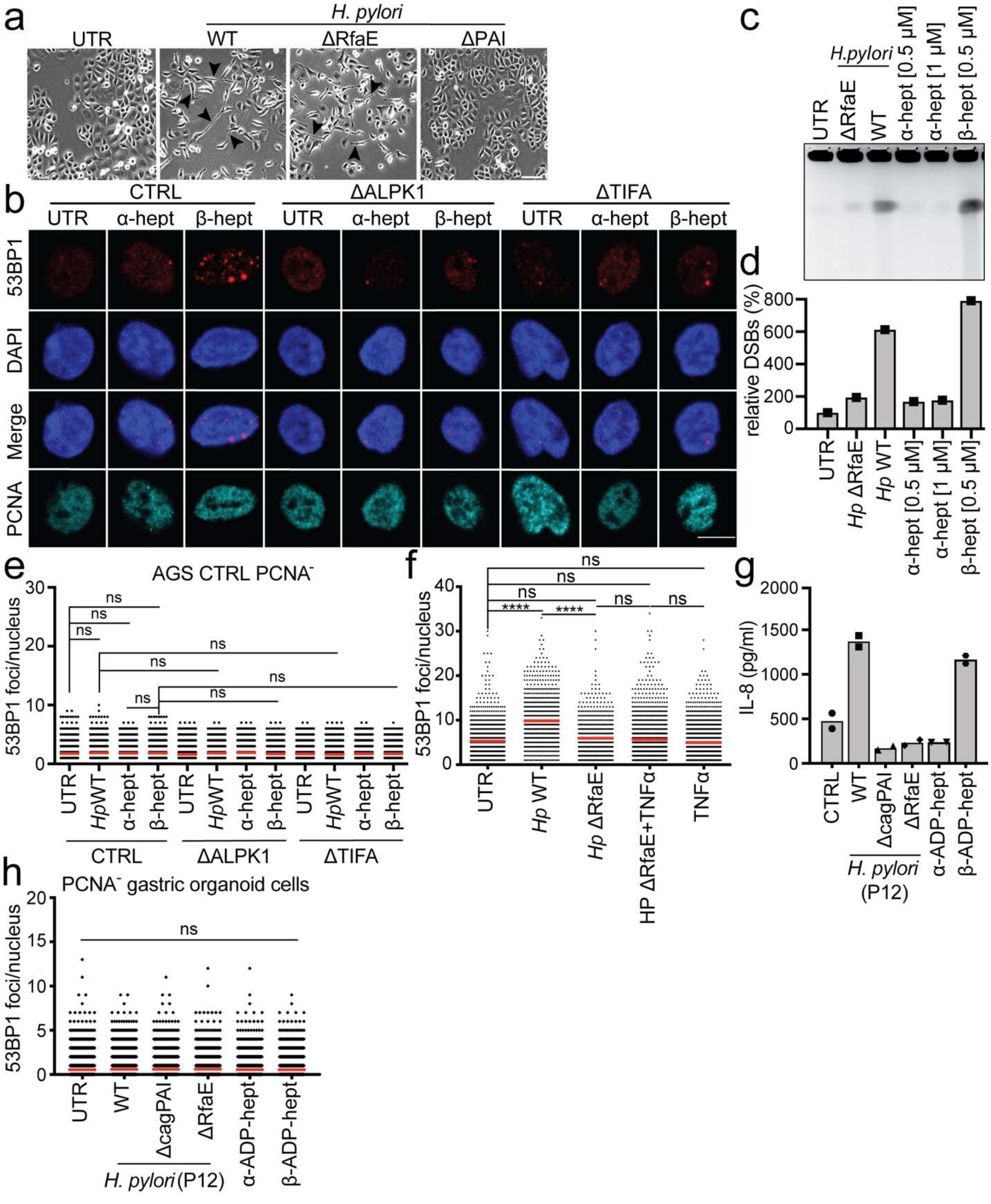

Supplementary Fig. 3. ADP-heptose-induced DNA DSBs are ALPK1- and TIFA-dependent and specific to S-phase cells as determined in cultured primary and transformed gastric epithelial cells. a AGS cells were infected for 6 hours with *H. pylori* P12 or its isogenic RfaE and Cag-PAI mutants at an MOI of 50 and examined by light microscopy for cell elongation and scattering as an indicator of CagA delivery. Arrows point to characteristic elongated cells. Images are representative of three independently

conducted experiments. Scale bar, 100 μm. **b** Wild type (CTRL), ALPK1-deficient (ΔALPK1) and TIFA-deficient (ΔTIFA) AGS cells were exposed to α- or β-ADP-heptose at 0.5 μM final concentration for 6 hours and subjected to immunofluorescence staining for 53BP1 and PCNA as well as DAPI. Images are representative of three independent experiments. Scale bar, 10 μm. **c,d** AGS cells were infected for 6 hours with *H. pylori* P12 or its isogenic RfaE mutant at an MOI of 50, or exposed to α- or β-ADP-heptose at the indicated final concentration for 6 hours and subjected to pulsed-field gel electrophoresis. The scan of the gel is shown in c alongside its quantification in d. **e** Wild type (CTRL), ALPK1-deficient (ΔALPK1) and TIFA-deficient (ΔTIFA) AGS cells were either infected for 6 hours with *H. pylori* P12 or exposed to α- or β-ADP-heptose at 0.5 μM final concentration for 6 hours and subjected to immunofluorescence staining for 53BP1 and PCNA as well as DAPI. Scatter dot plots of >793 and up to 1081 PCNA-negative cells per condition are shown. Data are pooled from three independent experiments. Red lines in e indicate medians. **f** AGS cells were infected for 6 hours with *H. pylori* P12 or its isogenic RfaE mutant at an MOI of 50, and/or treated with 10 nM TNF-α, and subjected to immunofluorescence staining for 53BP1 and PCNA as well as DAPI. Scatter dot plots of >604 and up to 1508 PCNA-positive cells per condition are shown. Data are pooled from three independent experiments. **g,h** Gastric organoids were transferred to 2D cultures and infected with the indicated strains of *H. pylori* P12 (MOI of 50) or exposed to α- or β-ADP-heptose at 0.5 μM final concentration for 6 hours. Supernatants were subjected to IL-8 ELISA and cells were subjected to immunofluorescence staining for 53BP1 and PCNA, as well as DAPI. IL-8 secretion is shown in g, and scatter dot plots of >1888 and up to 2760 PCNA-negative cells per condition are shown in h. Data in g and h are pooled from two independent experiments with cells derived from two different donors. Red lines indicate medians. P-values in e, f and h were calculated by one-way ANOVA with Dunn's multiple comparisons correction; ns, not significant; ****$p < 0.0001$. A statistical analysis of the two replicates per condition in g was not possible.

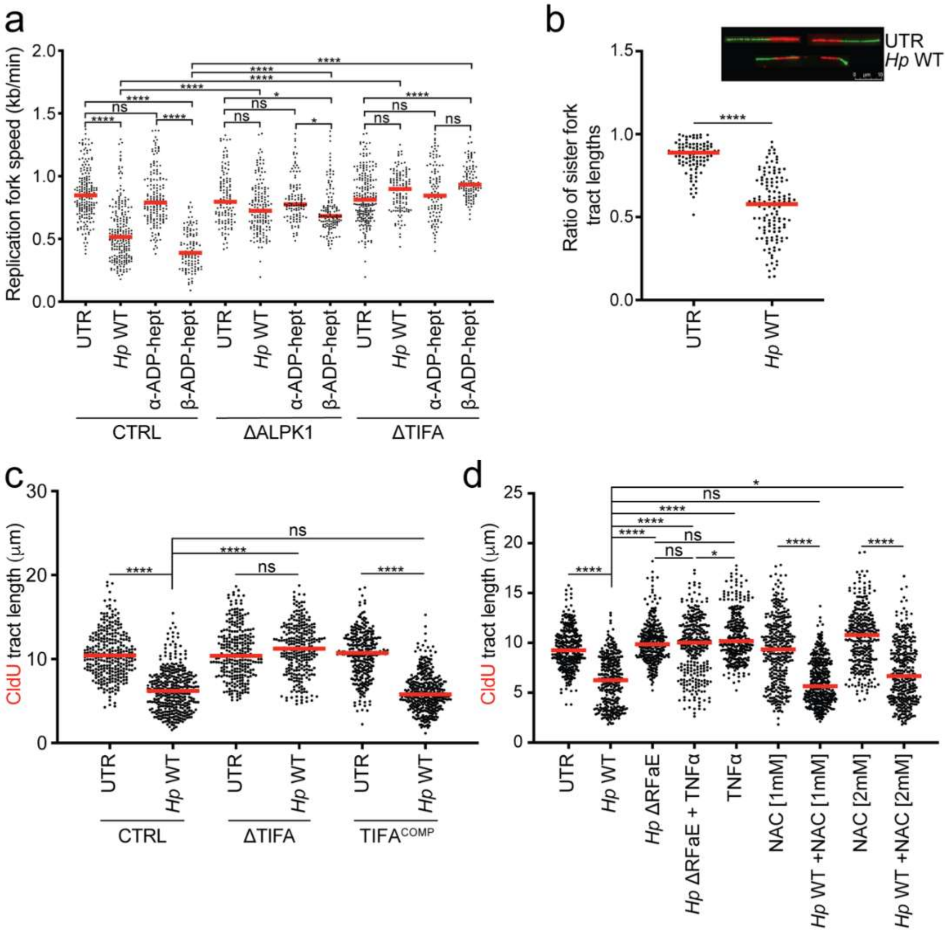

Supplementary Fig. 4. *H. pylori*-induced replication stress is RfaE-, ALPK1- and TIFA-dependent, but independent of reactive oxygen species. a Wild type (CTRL), ALPK1-deficient (ΔALPK1) and TIFA-deficient (ΔTIFA) AGS cells were either infected for 6 hours with *H. pylori* strain P12 (MOI of 50) or treated with 0.5 µM α- or β-ADP-heptose and then labeled sequentially with CIdU and IdU as shown in Figure 4a. Scatter dot plots of replication fork speed (in bp/min) were calculated based on the assumption that 1 µm of DNA fiber corresponds to 2.59 kb. At least 106 and up to 318 fibers were analyzed per condition as described in the legend to Figure 4c. Data in a are pooled from two independent experiments. **b** Sister fork asymmetry as determined by calculating ratios of CIdU tract lengths of DNA fibers generated from AGS cells that had been infected or not for 6 hours with *H. pylori* strain P12 (MOI of 50). CIdU/CIdU ratios were calculated by dividing the length of the shorter tract by the length of longer tract. At least 100 and up to 133 fibers were analyzed per condition. Data are pooled from two independent experiments. The inset shows representative sister forks of the two indicated conditions. **c** The indicated wild type and mutant cell lines in the AGS background were infected for 6 hours with *H. pylori* strain P12 at MOI 50 and subjected to the quantification of the length of newly synthesized DNA tracts. At least 281 and up to 386 fibers were analyzed per condition. Data are pooled from three independent experiments. **d** AGS cells were infected for 6 hours with *H. pylori* P12 or its isogenic RfaE mutant at an MOI of 50, and/or treated with 10 nM TNF-α; N-acetyl-cysteine (NAC) was added where indicated at 1 or 2mM final concentration. Cells were subjected to the quantification of the length of newly synthesized DNA tracts. Data in d are pooled from three independent experiments and at least 308 up to 380 fibers were analyzed per condition. Red lines indicate medians throughout. P-values in a-d were calculated by one-way ANOVA with Dunn's multiple comparisons correction; ns, not significant; *$p < 0.05$, ****$p < 0.0001$.

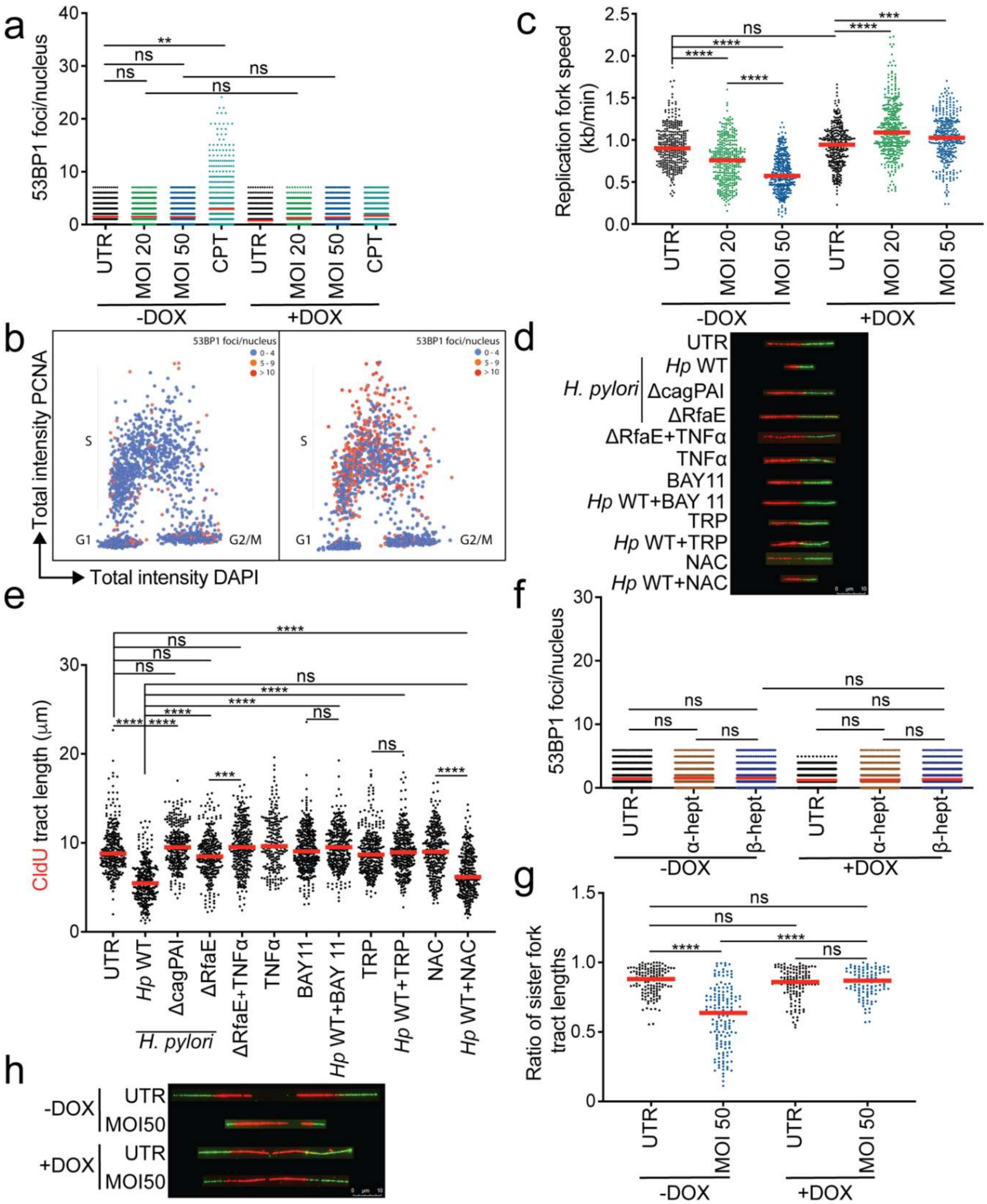

Supplementary Fig. 5. ***H. pylori*** **infection induces R-loop-mediated replication stress in U2OS cells. a** U2OS cells were either infected for 6 hours with *H. pylori* P12 (MOI of 20 or 50), or treated with 100 nM camptothecin (CPT), and were treated or not with doxycycline (-/+ DOX) to induce the expression of RNase H1. Cells were subjected to immunofluorescence staining for 53BP1 and PCNA as well as DAPI. Scatter dot plots of >500 and up to 800 PCNA-negative cells per condition are shown. Data are pooled from three independent experiments. **b** U2OS cells were infected for 6 hours with *H. pylori* P12 (MOI of 50) and subjected to immunofluorescence staining for 53BP1 and PCNA as well as DAPI. The PCNA and DAPI

signal intensities of ~800 uninfected and as many *H. pylori*-infected cells were plotted to visualize cell cycle phase (G1, S, G2/M). The color code indicates the number of 53BP1 foci/nucleus. **c** U2OS cells were infected and treated with doxycycline as described in a and additionally labeled sequentially with CIdU and IdU as shown in Fig. 4a for the assessment of DNA fiber tract length. Scatter dot plots of replication fork speed (in bp/min), as calculated based on the assumption that 1 μm of DNA fiber corresponds to 2.59 kb. At least 100 and up to 500 fibers were analyzed per condition. Data in c are pooled from three independent experiments. **d,e** Wild type U2OS cells were infected with the indicated strains of *H. pylori* (MOI of 50) and/or exposed to the NF-κB inhibitor BAY 11-7082 (1 μM) or triptolide (100 nM), or TNF-α (10 nM), and additionally labeled sequentially with CIdU and IdU for the assessment of replication tract length. Representative DNA fibers are shown in d and scatter dot plots of CIdU tract length (in μm) are shown in e for the indicated conditions. At least 200 and up to 500 fibers were analyzed per condition. Data in e are pooled from three independent experiments. **f** U2OS cells were exposed to α- or β-ADP-heptose at 0.5 μM final concentration for 6 hours, and were treated or not with doxycycline (-/+ DOX) to induce the expression of RNase H1. Cells were subjected to immunofluorescence staining for 53BP1 and PCNA as well as DAPI. Scatter dot plots of >500 and up to 800 PCNA-negative cells per condition are shown. Data are pooled from three independent experiments. **g,h** Sister fork asymmetry as determined by calculating ratios of CIdU tract lengths of DNA fibers generated from U2OS cells that had been infected or not for 6 hours with *H. pylori* strain P12 (MOI of 50) and treated or not with doxycycline. CIdU/CIdU ratios were calculated by dividing the length of the shorter tract by the length of longer tract. At least 102 and up to 155 fibers were analyzed per condition. Data in g are pooled from two independent experiments. Representative fibers are shown in h. Red lines indicate medians throughout. P-values in a, c, e, f and g were calculated by one-way ANOVA with Dunn's multiple comparisons correction; ns, not significant; $^{**}p < 0.01$, $^{***}p < 0.005$, $^{****}p < 0.0001$..

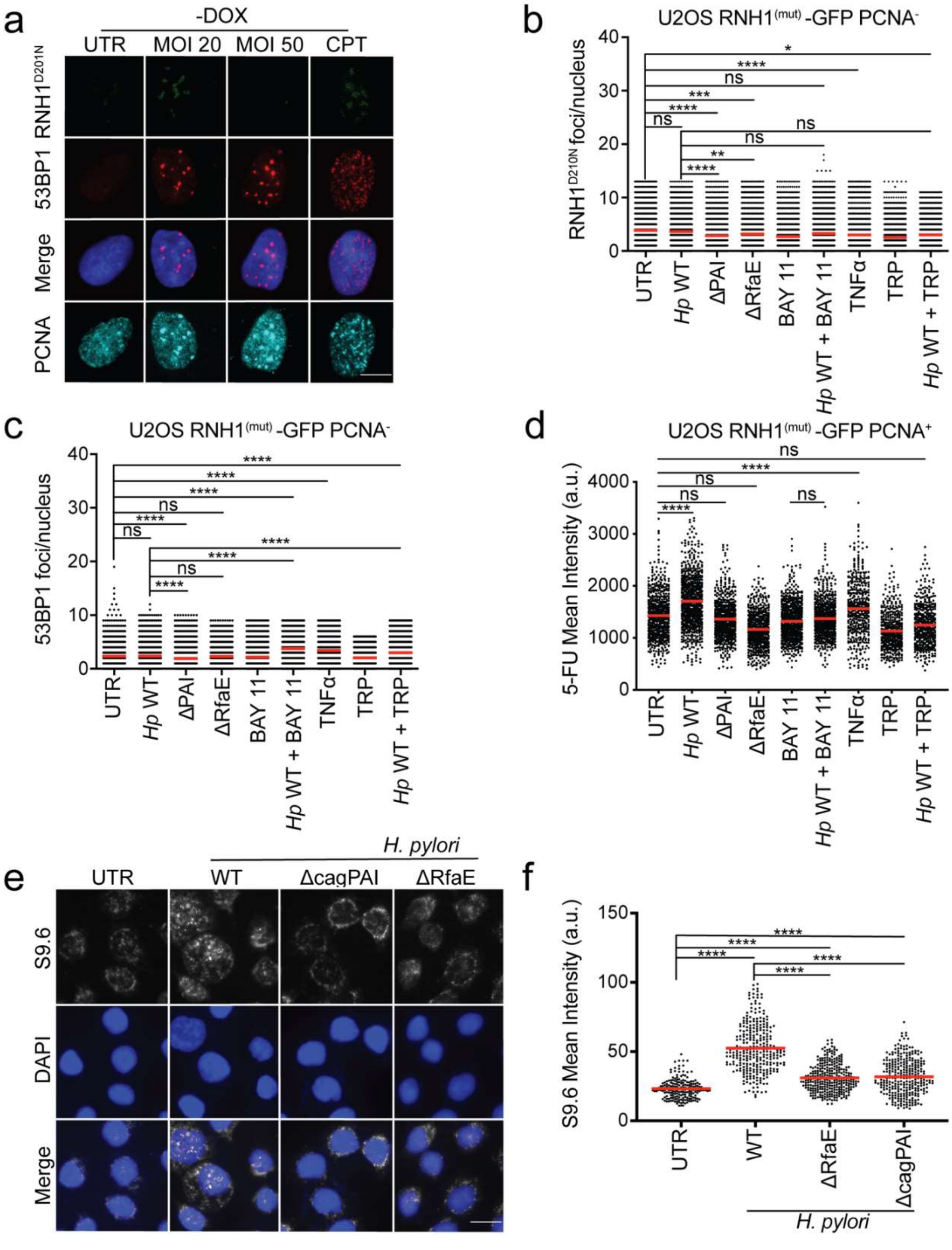

Supplementary Fig. 6. *H. pylori* infection induces R-loops in S-phase cells in an RfaE- and NF-kB-dependent manner. a U2OS cells were either infected for 6 hours with *H. pylori* P12 (MOI of 20 or 50), or treated with 100 nM camptothecin (CPT), and were treated or not with doxycycline (-/+ DOX) to induce the expression of a (D210N) mutant version of RNase H1 fused to GFP (RNH1^{D210N}). Cells were subjected to immunofluorescence staining for 53BP1 and PCNA as well as DAPI. Representative images are shown of the untreated (-DOX) condition, of RNH1^{D210N}/GFP foci and 53BP1 foci of a PCNA-positive cell each; images are representative of three independent experiments. Scale bar, 10 µm. **b,c** U2OS cells were infected

with the indicated strains of *H. pylori* (MOI of 50) and/or exposed to the NF-κB inhibitor BAY 11-7082 (1 μM) or triptolide (100 nM), or TNF-α (10 nM) and treated with doxycycline to induce the expression of RNH1^{D210N}/GFP. Cells were subjected to immunofluorescence staining for 53BP1 and PCNA as well as DAPI. Scatter dot plots of RNH1^{D210N}/GFP foci of >889 and up to 981 PCNA^{-} cells per condition are shown in b, and of 53BP1 foci of >931 and up to 1029 cells per condition in c. Data in b and c are pooled from three independent experiments. **d** U2OS cells were treated as described above, and additionally exposed to 1 μM 5-FU for the last hour of a 6-hour infection experiment. 5-FU mean fluorescence intensity was quantified by microscopy. A minimum of 436 and up to 748 cells were analyzed per condition. Representative data from one experiment of two is shown. **e,f** AGS cells were infected with the indicated strains of *H. pylori* (MOI of 50) for 6 hours and subjected to immunofluorescence staining for RNA/DNA hybrids (clone S9.6) and DAPI. Representative images are shown in e (scale bar 15 μm) and quantitative data from one representative experiment of two independently conducted ones is shown in f. At least 190 and up to 305 cells were analyzed per condition. Red lines indicate medians throughout. P-values in b, c, d and f were calculated by one-way ANOVA with Dunn's multiple comparisons correction; ns, not significant; *$p < 0.05$, **$p < 0.01$, ***$p < 0.005$, ****$p < 0.0001$.

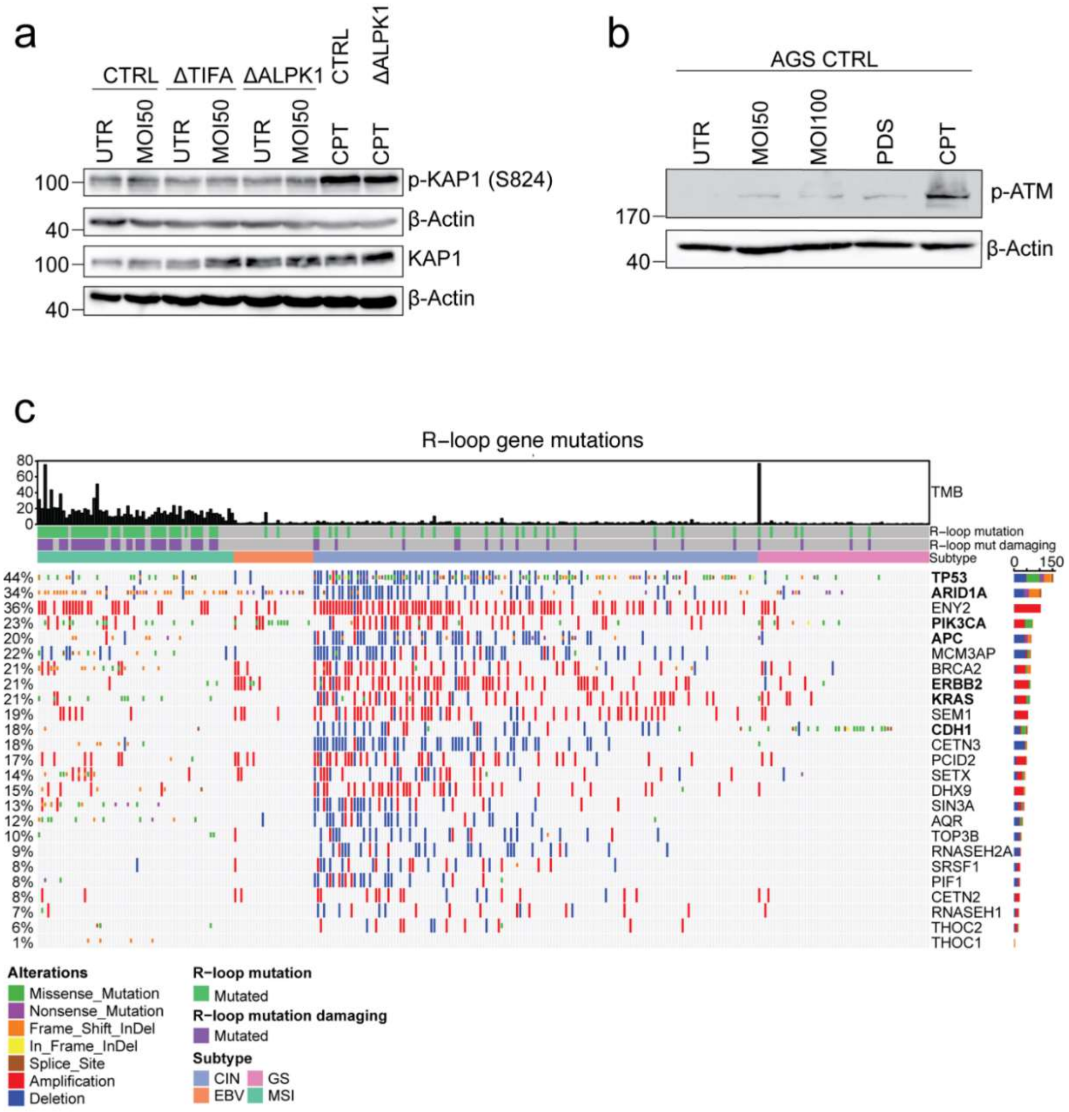

Supplementary Fig. 7. *H. pylori* induces DNA damage without an associated DNA damage response, and factors involved in R-loop metabolism are recurrently mutated in gastric cancer. a The indicated wild type and mutant cell lines in the AGS background were infected for 6 hours with *H. pylori* strain P12 at MOI 50 or treated with 100 nM camptothecin (CPT). Extracts were subjected to Western blotting using the indicated antibodies for total and phosphorylated KAP-1; β-actin was used as loading control. **b** AGS cells were infected for 6 hours with *H. pylori* strain P12 at MOI 50 or 100 or treated with 100 nM camptothecin (CPT) or 10 µm pyridostatin (PDS). Extracts were subjected to Western blotting using the indicated antibodies for phosphorylated ATM, with β-actin serving as loading control. Blots in a and b are representative of three independently conducted experiments. **c** The mutational landscape of gastric cancer with relation to genes involved in R-loop metabolism. 227 tumors from treatment-naïve gastric cancer patients that had previously been subjected to array-based somatic copy number analysis, whole-exome sequencing and array-based DNA methylation profiling as part of the Cancer Genome Atlas (TCGA) project were screened for mutations and copy number variations in 18 R-loop metabolism genes, which are plotted alongside the seven known gastric cancer driver genes *CDH1, APC, TP53, ARID1A, PIK3CA, KRAS* and *ERBB2* (shown in bold), and along with the mutational burden per megabase (TMB). The color codes indicate the four subtypes of gastric cancer, and the types of genetic alterations as well as the presence of (any, or damaging only) mutations in R-loop metabolism genes (in green and purple).

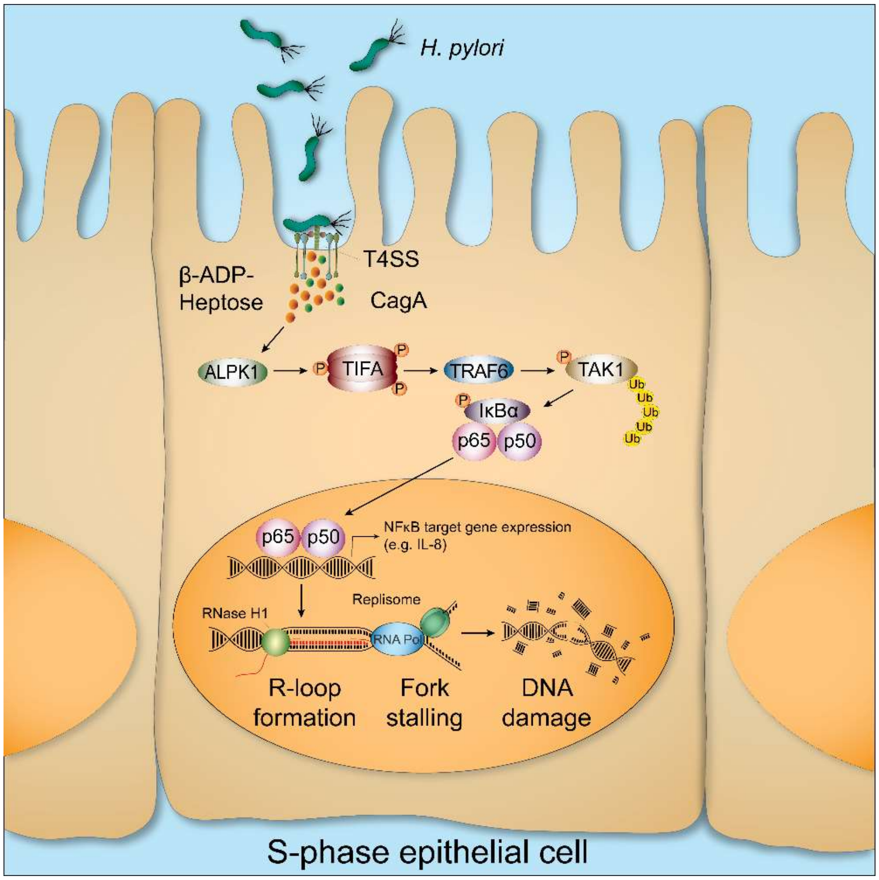

Supplementary Fig. 8. Schematic representation of *H. pylori*-induced R-loop formation and DNA damage. Upon attachment of *H. pylori* to gastric epithelial cells, the bacteria translocate ADP-heptose, HBP and/or other LPS biosynthetic intermediates into the cytoplasm of their target cells through the activity of the Cag-PAI-encoded T4SS. This process activates ALPK1, which phosphorylates TIFA and leads to the activation of TIFAsomes which, through activation of TAK1 kinase activity, promote IκBα degradation and the translocation of NF-κB to the nucleus. If NF-κB activation occurs in S-phase cells, active transcription machinery may collide with replication forks head-on, which may result in R-loop formation and replication fork stalling. Unless resolved promptly by dedicated enzymes including RNase H1, R-loop formation and the resulting replication stress may lead to DNA damage that manifests as DNA DSBs.
