# Supplementary material for: The ALPK1/TIFA/NF-κB axis links a bacterial carcinogen to R-loop-induced replication stress
Source: Nat Commun. 2020 Oct 9;11:5117. doi: 10.1038/s41467-020-18857-z (PMC7547021; doi:10.1038/s41467-020-18857-z)
Supplement: Supplementary file 3 — Description of Additional Supplementary Files [file 41467_2020_18857_MOESM3_ESM.docx]

**Legends to supplementary data files**

File Name: Supplementary Data 1

Description: Raw data of the experiments shown in Figure 1-7

File Name: Supplementary Data 2

Description: p- and n-values of all comparisons shown in main Figures 1-7 and suppl. Figures 1-7

File Name: Supplementary Data 3

Description: Raw data of the experiments shown in supplementary Figures 1-7

File Name: Supplementary Data 4

Description: Full scan image of the pulsed field gel shown in Figure 1D.

File Name: Supplementary Data 5

Description: Gating strategies used for the quantification of ROS-producing cells and Annexin V-positive apoptotic cells.

File Name: Supplementary Data 6

Description: Full scan image of the pulsed field gel shown in Figure 3D.

File Name: Supplementary Data 7

Description: Full scan image of the pulsed field gel shown in supplementary Figure 3C.

File Name: Supplementary Data 8

Description: Full scan image of the b-actin Western blot shown in supplementary Figure 7a

File Name: Supplementary Data 9

Description: Full scan image of the p-KAP1 Western blot shown in supplementary Figure 7a

File Name: Supplementary Data 10

Description: Full scan image of the b-actin and KAP1 Western blots shown in supplementary Figure 7a

File Name: Supplementary Data 11

Description: Full scan image of the b-actin Western blot shown in supplementary Figure 7b

File Name: Supplementary Data 12

Description: Full scan image of the p-ATM Western blot shown in supplementary Figure 7b

File Name: Supplementary Data 13

Description: supplementary table showing the 232 differentially mutated genes in *H. pylori*-positive and -negative gastric cancer
