# Supplementary figures and images for: The ALPK1/TIFA/NF-κB axis links a bacterial carcinogen to R-loop-induced replication stress
Source: Nat Commun. 2020 Oct 9;11:5117. doi: 10.1038/s41467-020-18857-z (PMC7547021; doi:10.1038/s41467-020-18857-z)

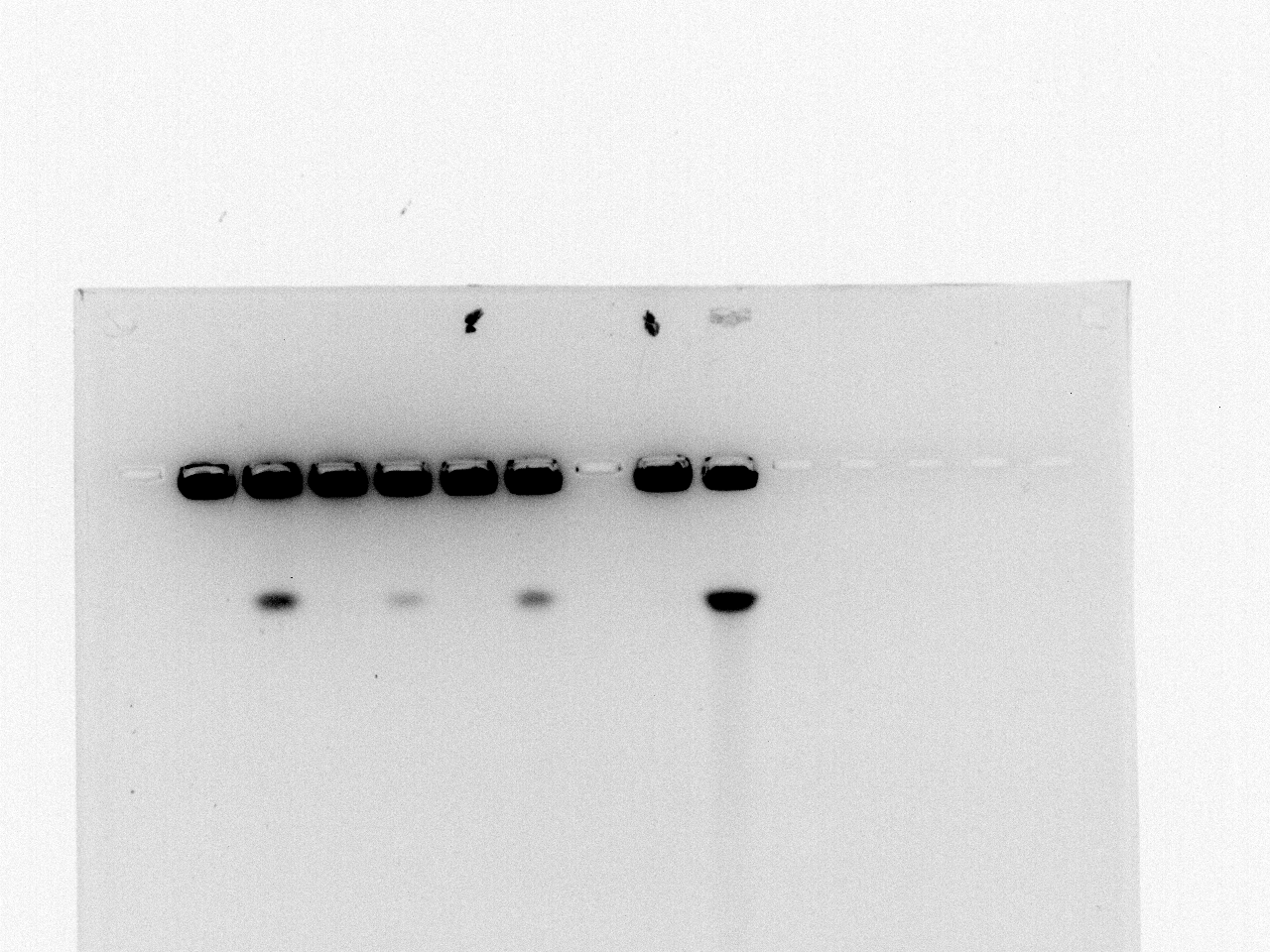

Supplement: Supplementary file 7 — Supplementary Data 4 [file 41467_2020_18857_MOESM7_ESM.tif]

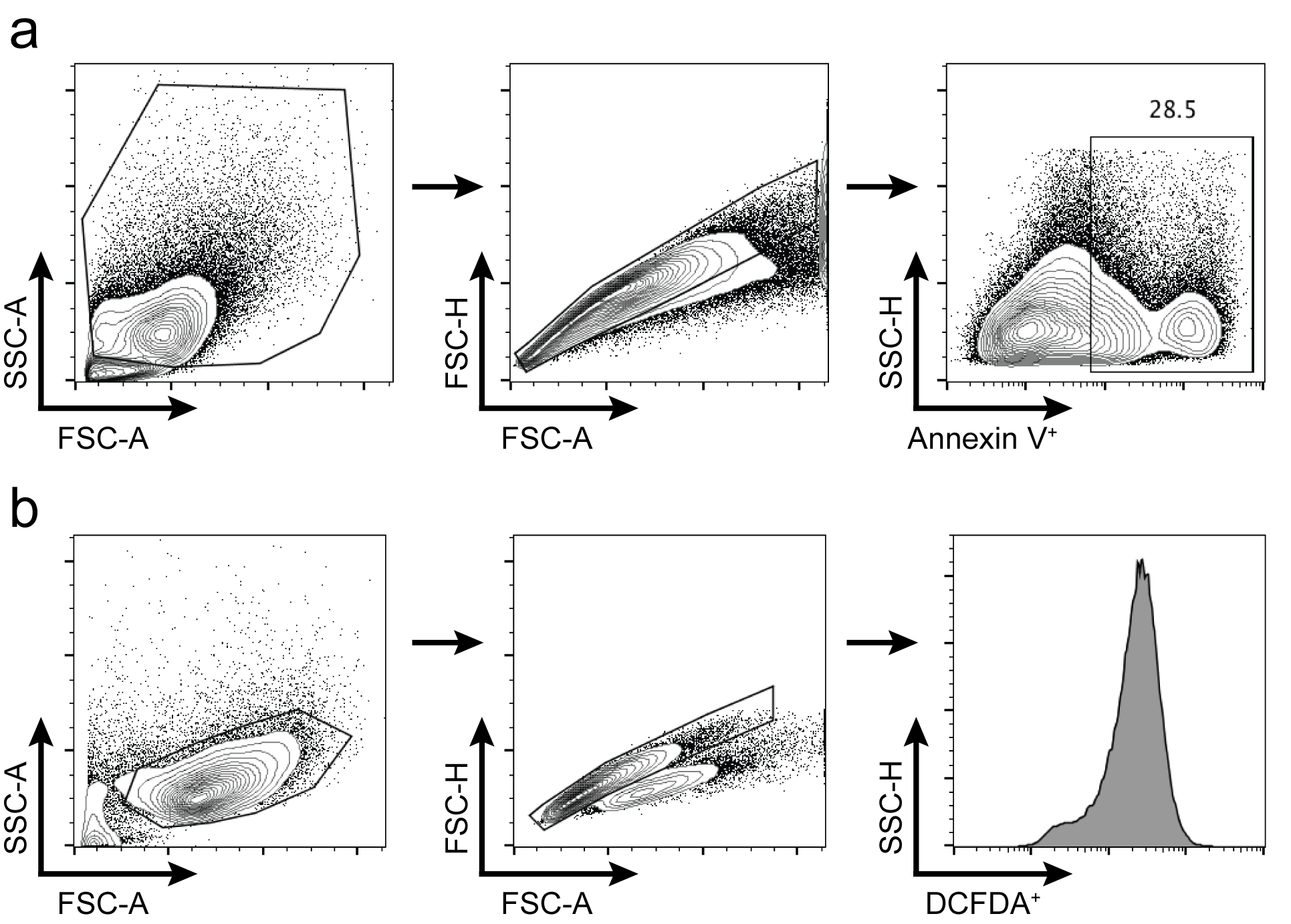

Supplement: Supplementary file 8 — Supplementary Data 5 [file 41467_2020_18857_MOESM8_ESM.tif]

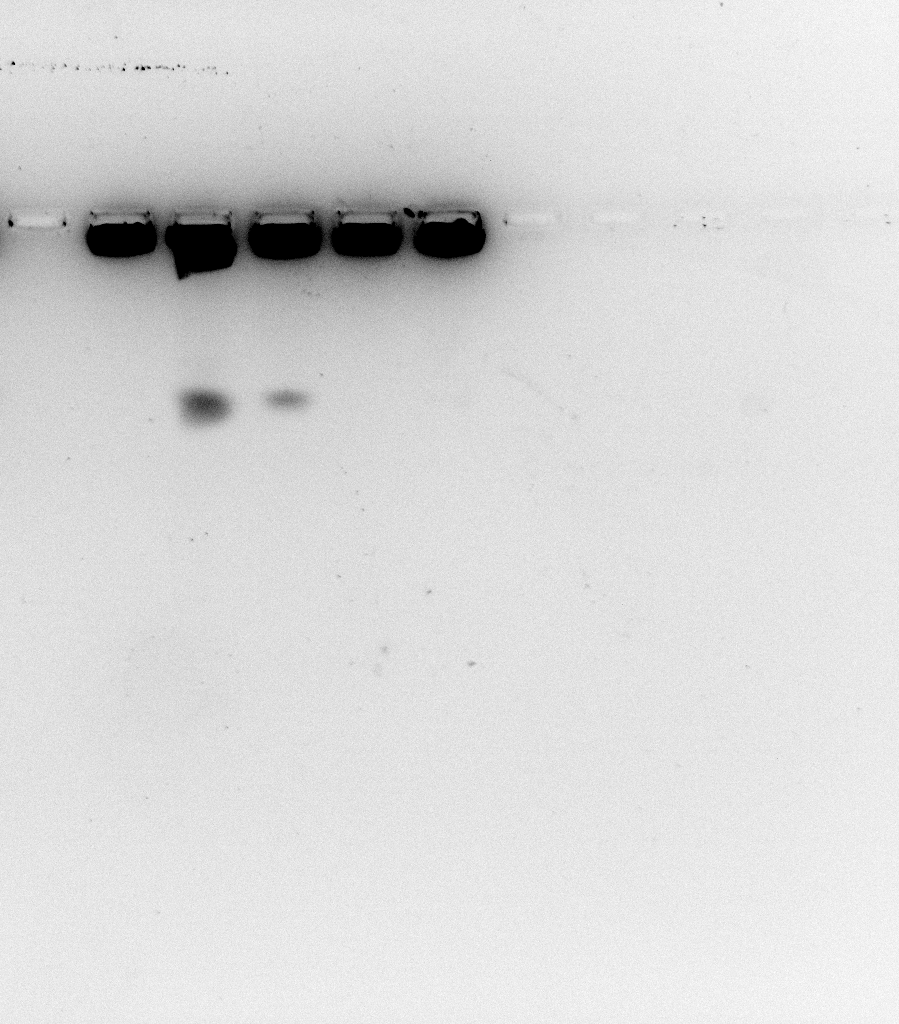

Supplement: Supplementary file 9 — Supplementary Data 6 [file 41467_2020_18857_MOESM9_ESM.tif]

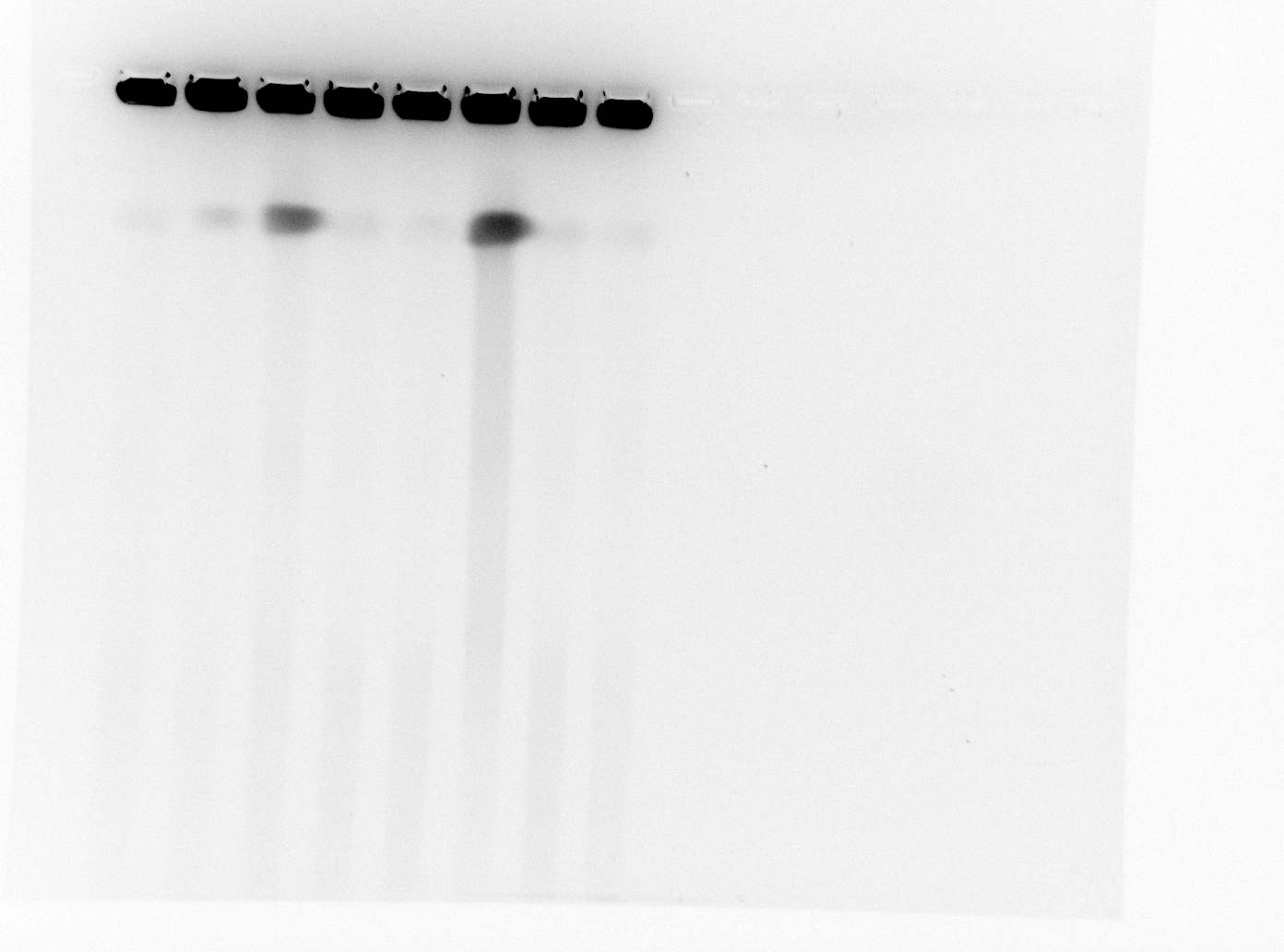

Supplement: Supplementary file 10 — Supplementary Data 7 [file 41467_2020_18857_MOESM10_ESM.tif]

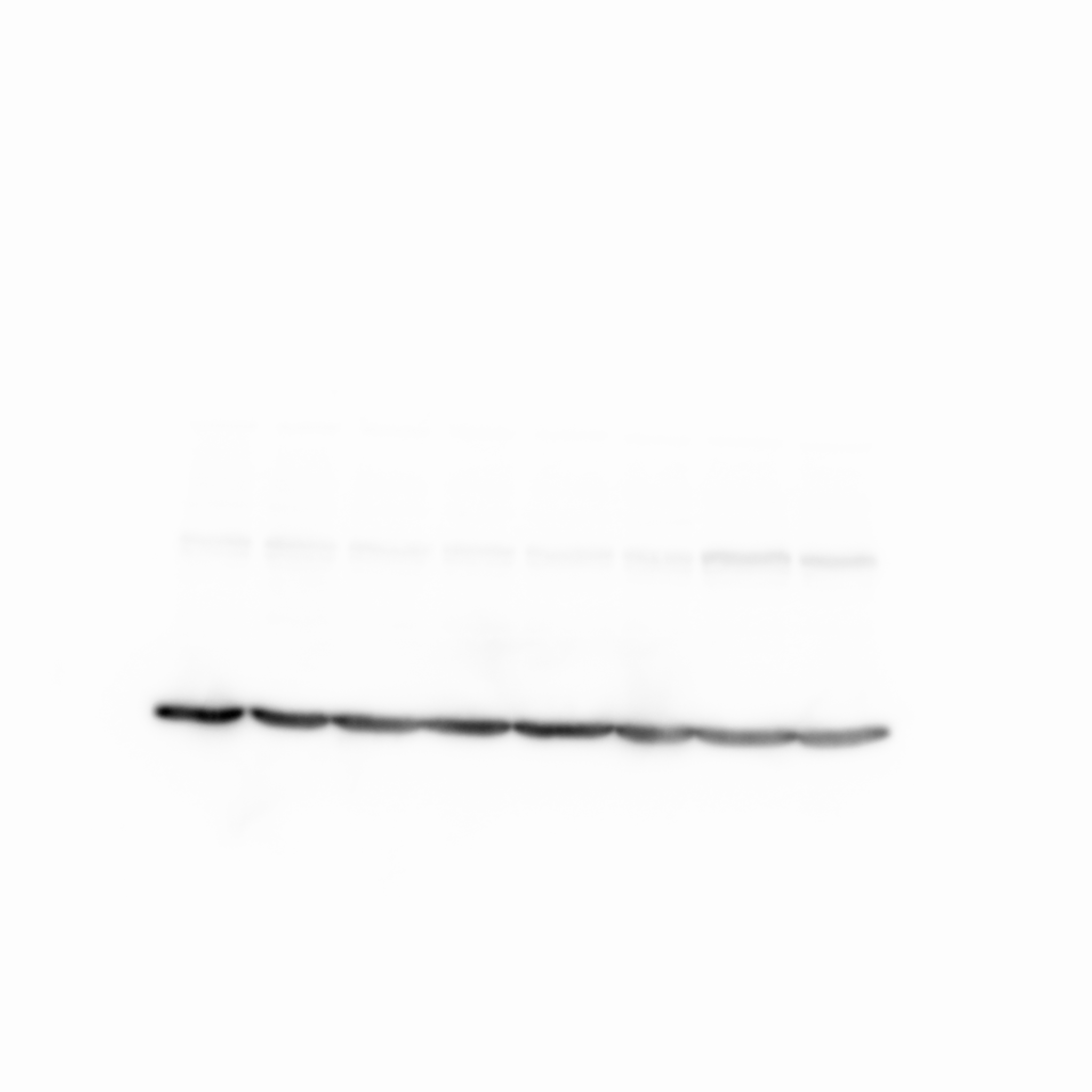

Supplement: Supplementary file 11 — Supplementary Data 8 [file 41467_2020_18857_MOESM11_ESM.tif]

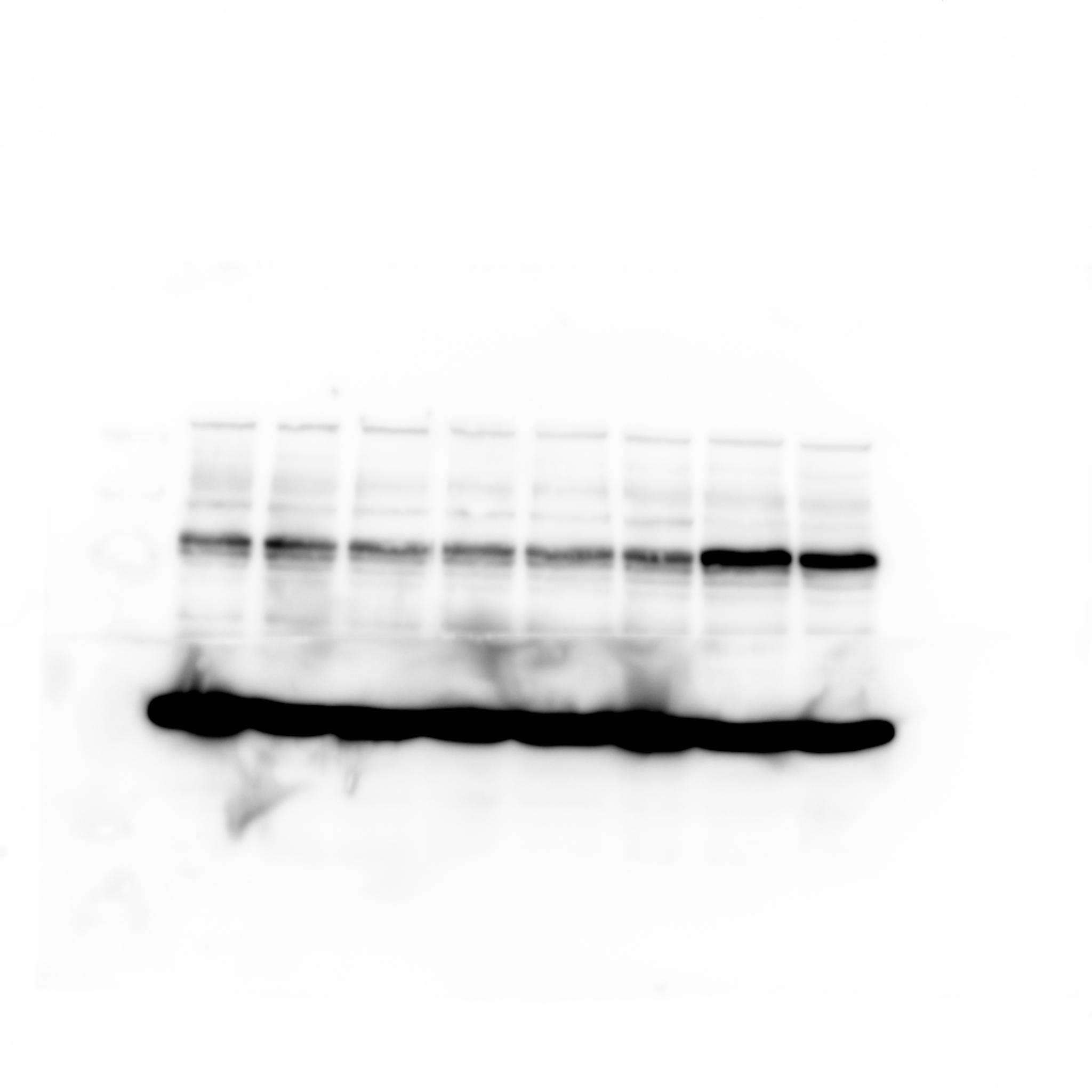

Supplement: Supplementary file 12 — Supplementary Data 9 [file 41467_2020_18857_MOESM12_ESM.tif]

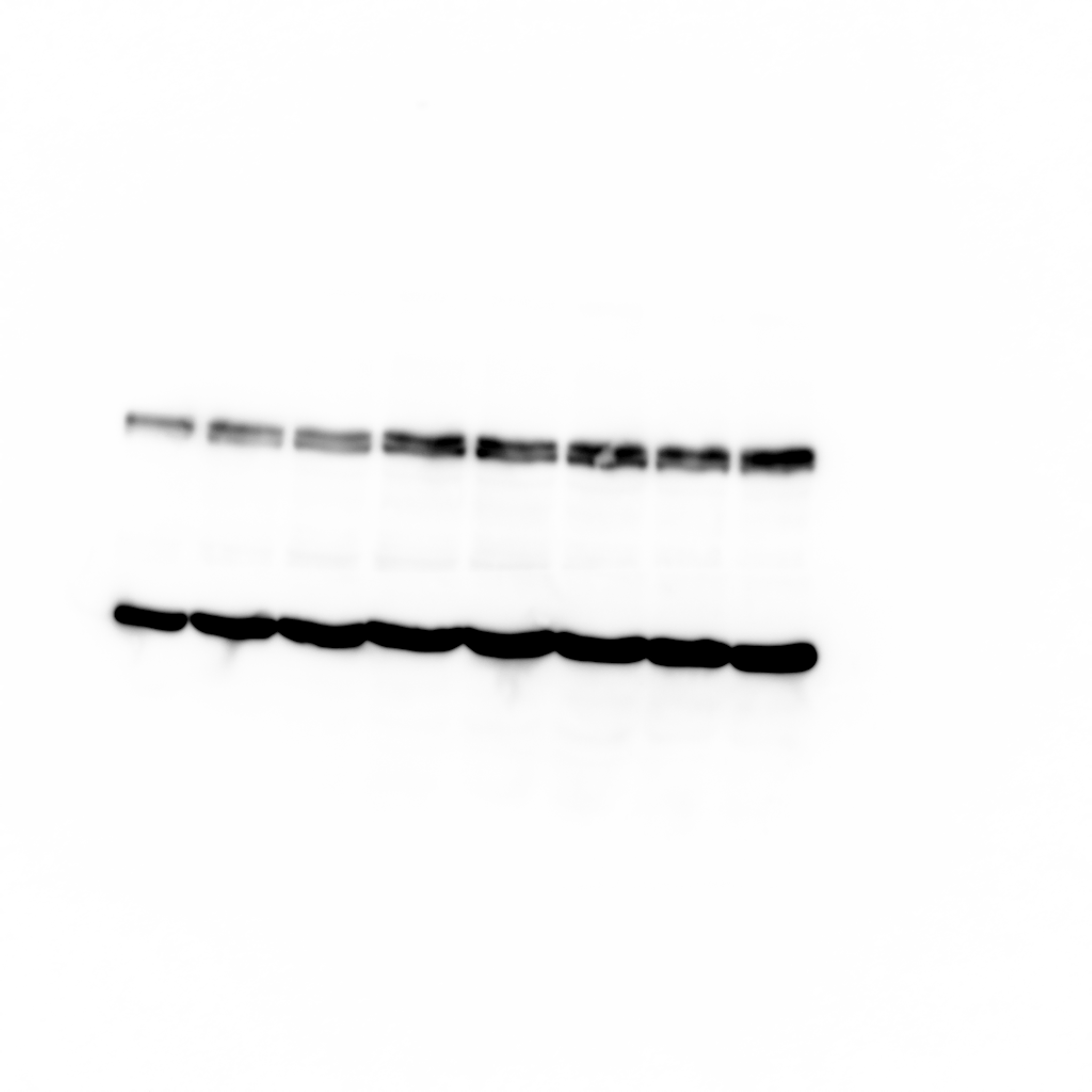

Supplement: Supplementary file 13 — Supplementary Data 10 [file 41467_2020_18857_MOESM13_ESM.tif]

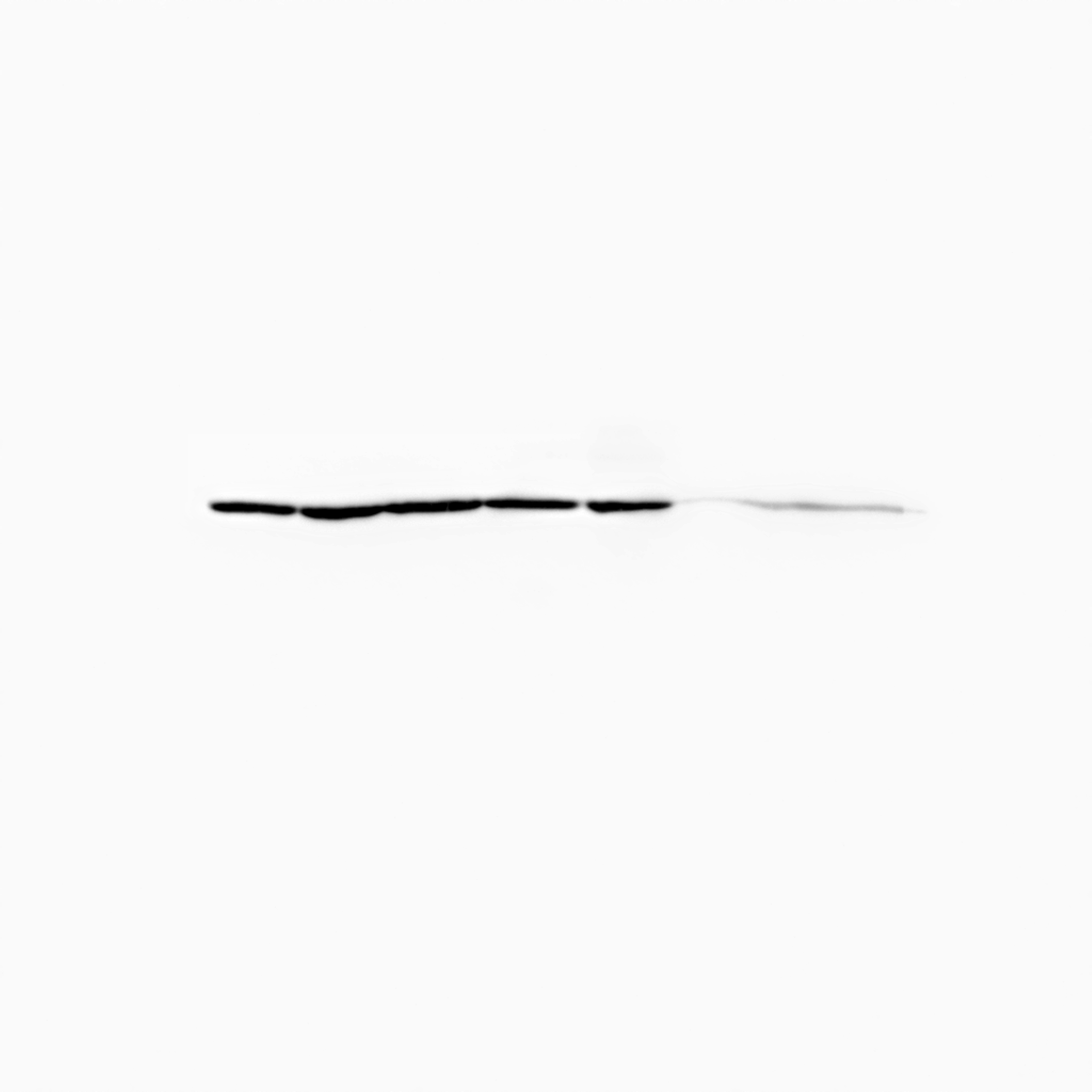

Supplement: Supplementary file 14 — Supplementary Data 11 [file 41467_2020_18857_MOESM14_ESM.tif]

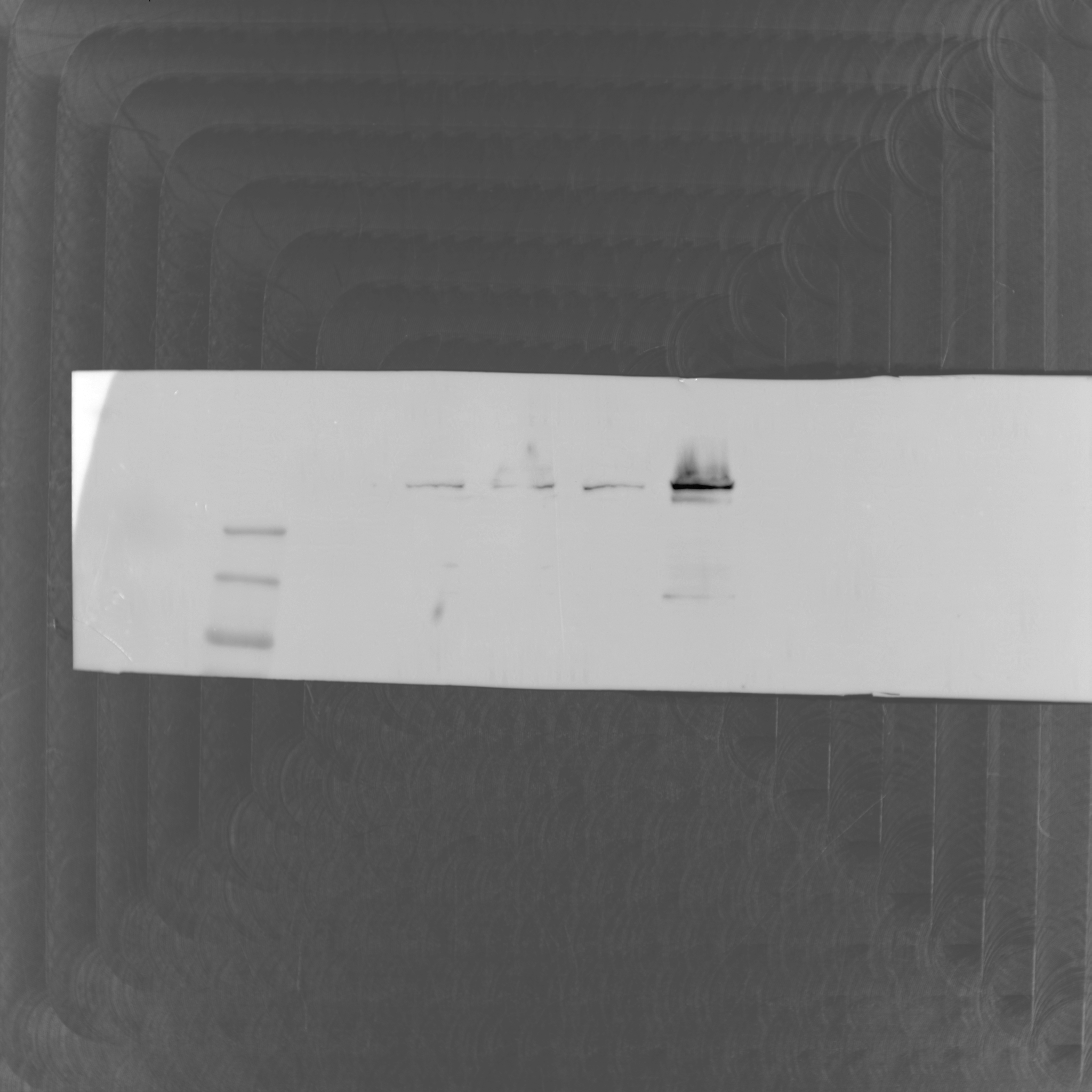

Supplement: Supplementary file 15 — Supplementary Data 12 [file 41467_2020_18857_MOESM15_ESM.tif]
