# Supplementary material for: The ALPK1/TIFA/NF-κB axis links a bacterial carcinogen to R-loop-induced replication stress
Source: Nat Commun. 2020 Oct 9;11:5117. doi: 10.1038/s41467-020-18857-z (PMC7547021; doi:10.1038/s41467-020-18857-z)
Supplement: Supplementary file 17 — Reporting Summary [file 41467_2020_18857_MOESM17_ESM.pdf]

nature research

Corresponding author(s): Anne Müller, Pavel Janscak

Last updated by author(s): Sep 1, 2020

Reporting Summary

Nature Research wishes to improve the reproducibility of the work that we publish. This form provides structure for consistency and transparency in reporting. For further information on Nature Research policies, see Authors & Referees and the Editorial Policy Checklist .

Statistics

For all statistical analyses, confirm that the following items are present in the figure legend, table legend, main text, or Methods section.

n/a	Confirmed	
☐	☒	The exact sample size (n) for each experimental group/condition, given as a discrete number and unit of measurement
☐	☒	A statement on whether measurements were taken from distinct samples or whether the same sample was measured repeatedly
☐	☒	The statistical test(s) used AND whether they are one- or two-sided<br>*Only common tests should be described solely by name; describe more complex techniques in the Methods section.*
☒	☐	A description of all covariates tested
☐	☒	A description of any assumptions or corrections, such as tests of normality and adjustment for multiple comparisons
☐	☒	A full description of the statistical parameters including central tendency (e.g. means) or other basic estimates (e.g. regression coefficient) AND variation (e.g. standard deviation) or associated estimates of uncertainty (e.g. confidence intervals)
☐	☒	For null hypothesis testing, the test statistic (e.g. F, t, r) with confidence intervals, effect sizes, degrees of freedom and P value noted<br>*Give P values as exact values whenever suitable.*
☒	☐	For Bayesian analysis, information on the choice of priors and Markov chain Monte Carlo settings
☒	☐	For hierarchical and complex designs, identification of the appropriate level for tests and full reporting of outcomes
☒	☐	Estimates of effect sizes (e.g. Cohen's d, Pearson's r), indicating how they were calculated

Our web collection on statistics for biologists contains articles on many of the points above.

Software and code

Policy information about availability of computer code

Data collection	Microscopy data collection was performed using ScanR imaging and analysis software (version 3.1).
Data analysis	Microscopy data analysis was performed using ScanR Analysis software (version 3.1) or CellProfiler (version 3.1.5.), or the software Fiji Image J (Version 2.0.0-rc-59/1.51j) for fiber analyses. IHC images were analysed using NDP.view (version 2). Data analysis was done in Prism (version 8.1.2.); FACS data were analyzed by Flowjo (version 10.4.2.). All packages are commercially available.

For manuscripts utilizing custom algorithms or software that are central to the research but not yet described in published literature, software must be made available to editors/reviewers. We strongly encourage code deposition in a community repository (e.g. GitHub). See the Nature Research guidelines for submitting code & software for further information.

Data

Policy information about availability of data

All manuscripts must include a data availability statement. This statement should provide the following information, where applicable:

- Accession codes, unique identifiers, or web links for publicly available datasets
- A list of figures that have associated raw data
- A description of any restrictions on data availability

There are no restrictions to data availability associated with this manuscript. All raw data will be made publicly available as per Nature Communications specifications. We are now including the following data availability section in the manuscript:
Data Availability
The source data underlying Figs 1-7 and Supplementary Figs 1-7 are provided as a Source Data file. All the other data supporting the findings of this study are available within the article and its supplementary information files . A reporting summary for this article is available as a Supplementary Information file.

Field-specific reporting

Please select the one below that is the best fit for your research. If you are not sure, read the appropriate sections before making your selection.

- [x] Life sciences
- [ ] Behavioural & social sciences
- [ ] Ecological, evolutionary & environmental sciences

For a reference copy of the document with all sections, see nature.com/documents/nr-reporting-summary-flat.pdf

Life sciences study design

All studies must disclose on these points even when the disclosure is negative.

Sample size	Every experiment that is shown in our manuscript has been repeated at least three times, and pooled data from at least three independent experiments are shown throughout. The organoid experiments were performed on two independent donor organoids. We chose sample sizes of several hundred to one thousand cells/fibers per experiment; with an effect size of factor 5-10 fold increases in 53BP1 foci per nucleus, or RNAse H1 foci per nucleus, this number of cells/fibers analyzed (automated image analysis was performed throughout) has proved to give highly meaningful differences and statistically significant results. Most of our p-values are extremely low (<0.001) despite the need for multiple comparison corrections in virtually all graphs (we are always comparing at least four, and up to 10 different conditions per graph).
Data exclusions	No data were excluded from any of the analyses.
Replication	All reported findings were extraordinarily robust. Only findings that replicated at least three times were included.
Randomization	This work includes no model systems to which randomization would apply; it makes no sense to randomize transformed cell lines, which grow very uniformly, to experimental conditions. We paid attention that 2D organoid cultures of comparable density and viability were assigned to the experimental conditions that applied in that experiment (infection with WT and mutant H. pylori strains, treatment with ADP heptoses). All conditions were pre-determined, three technical replicates were included throughout, and at least three independent experiments were conducted.
Blinding	not applicable, as the experimenter usually handled cells and organoids from the beginning of an experiment to its end; one exception was the data analysis of 53BP1-stained cell cultures, which were -in some instances- recorded and analyzed by two different experimenters (the first authors); wherever possible, the data analysis was done blindly by the second experimenter without knowledge of the identity of the samples.

Reporting for specific materials, systems and methods

We require information from authors about some types of materials, experimental systems and methods used in many studies. Here, indicate whether each material, system or method listed is relevant to your study. If you are not sure if a list item applies to your research, read the appropriate section before selecting a response.

Materials & experimental systems

n/a	Involved in the study	
☐	☒	Antibodies
☐	☒	Eukaryotic cell lines
☒	☐	Palaeontology
☒	☐	Animals and other organisms
☐	☒	Human research participants
☒	☐	Clinical data

Methods

n/a	Involved in the study	
☒	☐	ChIP-seq
☐	☒	Flow cytometry
☒	☐	MRI-based neuroimaging

Antibodies

Antibodies used	For immunofluorescence microscopy, the following primary antibodies and dilutions were used: anti-phospho Histone H2A.X (Ser139) mouse monoclonal (Millipore, order number 05-636-AF647, clone JBW301, 1:300), anti-53BP1 rabbit polyclonal (Santa Cruz, sc-22760, 1:300), and anti-PCNA Mouse monoclonal (Santa Cruz, sc56, PC10, 1:250). For IHC, the following antibodies were used: Ki-67 rabbit monoclonal antibody (clone 30-9, order number not applicable, Ventana Roche, 1:100), anti-H. pylori polyclonal rabbit antibody (B0471, 3382898-b0471, Dako, 1:100). Secondary antibodies and dilutions were: Alexa Fluor 488 Goat Anti-Rabbit IgG (Life Technologies, A11034, 1:400), Alexa Fluor 568 Goat Anti-Rabbit (Life Technologies, A11036, 1:400), Alexa Fluor 568 Goat Anti-Mouse (Life Technologies, A11031, 1:400), Alexa Fluor 647 Goat Anti-Rabbit (Life Technologies, A21245, 1:400), Alexa Fluor 647 Goat Anti-Mouse (Invitrogen, A21235, 1:400).
Validation	All our antibodies were purchased from commercial vendors and were extensively validated regarding species specificity, cross-reactivity with other antigens, and their use in various applications. This information is readily available on the supplier websites.<br>Validation of primary antibodies: Anti-phospho-Histone H2A.X (Ser139), clone JBW301 is a well published Mouse Monoclonal Antibody validated in ChIP, ICC, IF, WB. This purified mAb is highly specific for phospho-Histone H2A.X (Ser139) also known as H2AXS139p. The anti-53BP1 antibody was validated for binding to an epitope corresponding to amino acids 1-300 mapping at

	the N-terminus of 53BP1 of human origin by the supplier. The PCNA antibody (PC10) has been validated by the supplier and recommended for detection of PCNA p36 protein expressed at high levels in proliferating cells of mouse, rat, human, insect and S.pombe origin by Western Blotting, immunoprecipitation, immunofluorescence, immunohistochemistry and flow cytometry. The Ki67 antibody is intended for, and validated for use to identify stained proliferating cells by light microscopy in sections of formalin-fixed, paraffin-embedded tissue on a Ventana automated slide stainer.

Eukaryotic cell lines

Policy information about cell lines

Cell line source(s)	ATCC: AGS (ATCC® CRL-1739) and U2OS (ATCC® HTB-96)
Authentication	The two cell lines were directly obtained from, and authenticated by the commercial source ATCC. The AGS cell line was originally derived from fragments of a tumor resected from a patient who had received no prior therapy. This is a hyperdiploid human cell line. The modal chromosome number was 49, occurring in 60% of cells. The rate of polyploidy was 3.6%. Single copy each for der(8)t(1;8) (q12;p23), der(19)t(19;?) (q13.6;?), minute chromosome M3, and C-group-like M12 was seen in all cells. The cell line U-2 OS is chromosomally highly altered, with chromosome counts in the hypertriploid range. Very few normal chromosomes are present, but a high number of stable marker chromosomes are identified. Different chromosomal rearrangements involving the same chromosomes (N1, N7, N9, and N11 particularly), are seen. Twenty-two markers are found including: t(9qter--->9q21::1p36--->1p::?), 7p+, iso(17q), t(15q;?), 4q+, del(3)(q21), 5q(aberrant) and others.
Mycoplasma contamination	We routinely do mycoplasma testing on our cell lines, and have not had a mycoplasma-contaminated culture in >7 years.
Commonly misidentified lines (See ICLAC register)	No easily misidentified cell lines were used in this study. Quite to the contrary, both AGS and U2OS cells are widely used in experimental research of H. pylori infection and DNA damage, respectively.

Human research participants

Policy information about studies involving human research participants

Population characteristics	Formalin-fixed paraffin-embedded (FFPE) specimens of six patients with gastritis or normal controls from the Department of Pathology and Molecular Pathology Zurich were analyzed. The study was approved by the Cantonal Ethics Committee of Zurich (KEK).
Recruitment	Patient samples were selected based on their histological diagnosis of gastritis, and matched with three patients with normal gastric mucosa.
Ethics oversight	The study was approved by the Cantonal Ethics Committe of Zurich (KEK).

Note that full information on the approval of the study protocol must also be provided in the manuscript.

Flow Cytometry

Plots

Confirm that:

- [x] The axis labels state the marker and fluorochrome used (e.g. CD4-FITC).
- [x] The axis scales are clearly visible. Include numbers along axes only for bottom left plot of group (a 'group' is an analysis of identical markers).
- [x] All plots are contour plots with outliers or pseudocolor plots.
- [x] A numerical value for number of cells or percentage (with statistics) is provided.

Methodology

Sample preparation	For quantification of reactive oxygen species, AGS cells were stained with 25 µM 2',7' –dichlorofluorescin diacetate (DCFDA, Abcam, ab113851) for 6 hours. Cells were harvested by centrifugation, resuspended in 1x PBS/1%BSA and subjected to flow cytometry. Apoptosis was quantified by staining with PE-Cy7-labbeled AnnexinV (BioLegend, Cat No 559934) diluted in 1x AnnexinV Binding buffer (BD Bioscience, Cat No 640950) for 15 min at RT in the dark.
Instrument	Data was acquired on a CyAn ADP (Beckman Coulter) flow cytometer.
Software	Data were analyzed with the FlowJo software (version 10.4.2., TreeStar).
Cell population abundance	Sorting does not apply here. We quantified the relevant populations as % of parent population as shown in the submitted gating strategy.

Gating strategy

The gating strategy was applied based on unstained samples and based on positive and negative controls. Please see the attached gating strategy. Both protocols were simple (one color only), no compensation was required and the borders between positive and negative cells were straightforward to set based on the unstained control sample.

☒ Tick this box to confirm that a figure exemplifying the gating strategy is provided in the Supplementary Information.
